# Gray Matter Abnormalities in Temporal Lobe Epilepsy: Relationships with Resting-State Functional Connectivity and Episodic Memory Performance

**DOI:** 10.1371/journal.pone.0154660

**Published:** 2016-05-12

**Authors:** Gaelle E. Doucet, Xiaosong He, Michael Sperling, Ashwini Sharan, Joseph I. Tracy

**Affiliations:** 1 Department of Neurology, Thomas Jefferson University, Philadelphia, PA, United States of America; 2 Department of Neurosurgery, Thomas Jefferson University, Philadelphia, PA, United States of America; University of Modena and Reggio Emilia, ITALY

## Abstract

Temporal lobe epilepsy (TLE) affects multiple brain regions through evidence from both structural (gray matter; GM) and functional connectivity (FC) studies. We tested whether these structural abnormalities were associated with FC abnormalities, and assessed the ability of these measures to explain episodic memory impairments in this population. A resting-state and T1 sequences were acquired on 94 (45 with mesial temporal pathology) TLE patients and 50 controls, using magnetic resonance imaging (MRI) technique. A voxel-based morphometry analysis was computed to determine the GM volume differences between groups (right, left TLE, controls). Resting-state FC between the abnormal GM volume regions was computed, and compared between groups. Finally, we investigated the relation between EM, GM and FC findings. Patients with and without temporal pathology were analyzed separately. The results revealed reduced GM volume in multiple regions in the patients relative to the controls. Using FC, we found the abnormal GM regions did not display abnormal functional connectivity. Lastly, we found in left TLE patients, verbal episodic memory was associated with abnormal left posterior hippocampus volume, while in right TLE, non-verbal episodic memory was better predicted by resting-state FC measures. This study investigated TLE abnormalities using a multi-modal approach combining GM, FC and neurocognitive measures. We did not find that the GM abnormalities were functionally or abnormally connected during an inter-ictal resting state, which may reflect a weak sensitivity of functional connectivity to the epileptic network. We provided evidence that verbal and non-verbal episodic memory in left and right TLE patients may have distinct relationships with structural and functional measures. Lastly, we provide data suggesting that in the setting of occult, non-lesional right TLE pathology, a coupling of structural and functional abnormalities in extra-temporal/non-ictal regions is necessary to produce reductions in episodic memory recall. The latter, in particular, demonstrates the complex structure/function interactions at work when trying to understand cognition in TLE, suggesting that subtle network effects can emerge bearing specific relationships to hemisphere and the type of pathology.

## Introduction

There is growing body of evidence that brain abnormalities in focal epilepsies such temporal lobe epilepsy (TLE) are not limited to the epileptogenic region, but extend into widespread areas of the whole brain. These extra-temporal abnormalities have emerged from gray matter (GM)[1], white matter[2,3], metabolic[4,5], and, more recently, resting-state fMRI investigations (see review by [6]). In terms of structural abnormalities, voxel-based morphometry (VBM) studies in TLE have been effective in demonstrating reduced GM volume in multiple brain regions such as the mesial temporal lobe, thalami, insula, or sensory motor cortex[1,7,8]. Using this approach, differences between right and left TLE patients[8,9] and between TLE patients with and without mesial temporal sclerosis (MTS)[10,11] have been established. These extra-temporal structural abnormalities are considered to reflect the impact of seizure spread or epileptogenesis, providing a potential map or clue to the reaches of the epileptiform network emerging from the epileptogenic mesial temporal lobe[12,13]. Using a more direct assessment of brain network abnormalities, resting-state functional connectivity (FC) studies in TLE patients have demonstrated both temporal and extra-temporal functional connectivity abnormalities[14–16].

While these studies suggest that TLE preferentially affects a network of regions that are functionally and anatomically connected to the hippocampus[1,17–19], the strength of the association between structural (GM) and functional (FC) abnormalities remains untested. To our knowledge, only a limited number of studies have explored the relationship between GM concentration and FC in TLE patients [20,21]. Holmes et al. (20) investigated left TLE and found that regions with significantly reduced GM concentration correlated with change in resting FC involving the left hippocampus or left thalamus, with the nature of the change (increase or decrease) varying by region. McCormick et al. [21] found a focal GM loss in the epileptic temporal lobe related to two specific patterns of DMN abnormality, namely, decreased FC between the medial TL’s and posterior DMN, but increased intrahemispheric (anterior-posterior) FC. A complete understanding of these potential epileptic networks emerging from GM abnormalities requires measuring there impact on brain output and performance by including measures of neurocognitive functioning. Indeed, neurocognitive deficits are well-described in TLE[22], but the relation of those deficits to both structural and FC impairments, particularly those outside the epileptogenic temporal lobe is quite unclear. In fact, the number of studies showing a relation between GM volume and neurocognitive scores in TLE is quite limited [21,23,24]. Focke et al.[23] found that global GM volume correlated with neurocognitive scores in left TLE only. While McCormick et al. [21] revealed that the sections of the DMN noted above, were related to episodic memory, McCormick did not test for a relationship between the global (i.e., extra-temporal) structural integrity of the brain and FC. Also, Bonilha et al. [24] suggested that both general and specific verbal memory impairments in left TLE are associated with atrophy of the left mesial temporal lobe, including the hippocampus, the entorhinal, and the perirhinal cortex. This study, however, did not examine relationships with FC. Regarding resting-state FC, episodic memory deficits have been also correlated with FC abnormalities emerging from the hippocampus in both left and right TLE patients [14,21]. It is unknown, however, whether these episodic memory deficits are related to the abnormal FC emerging from the hippocampus, hippocampal atrophy, or a combination of the two. Accordingly, to date, data regarding the relation between extra-temporal GM and resting-state FC integrity is limited, and, more importantly, the degree to which that association impacts neurocognition in unilateral TLE patients has not been studied.

In this context, we sought to investigate structural (GM) abnormalities and their relation to resting-state FC and episodic memory performance in unilateral mesial and non-mesial TLE patients. To do so, we studied a large sample of 94 TLE patients and 50 healthy matched controls. Each participant underwent a MRI scan including a high-dimensional T1 sequence and a resting-state condition. Patients’ verbal and non-verbal episodic memory performances were also evaluated. Based on the regional GM abnormalities in TLE established through a VBM approach, we explored the integrity of resting-state FC emerging from and between these regions. We then went on to investigate whether these structural and functional measures were associated with episodic memory performance in the patients, examining as well the role played by MTS and the side of pathology in our findings. To our knowledge, no study has yet combined structural measures of macrostructural GM abnormalities with measures of resting-state FC integrity to test for, one, the presence of potential epileptic networks outside the temporal lobe, and, two, the impact of GM abnormality-based FC measures on neurocognitive measures such as episodic memory.

We hypothesized that the regions identified with reduced GM volume will form a network, as verified by resting-state FC, with individuals showing the strongest FC abnormalities showing the most impaired episodic memory. In addition, we expected that the strength of the relation between our structural and functional measures will predict the degree of episodic memory impairment in TLE, with this relationship varying as a function of memory material (e.g. verbal and non-verbal performance will be related to abnormalities in the left and right TLE, respectively). Our goal is to understand the functional impact of GM abnormalities in TLE, seeking to clarify the neuroplastic responses generated by TLE pathology both in terms of altering resting-state FC and cognitive skills (i.e., episodic memory).

## Materials and Methods

### Participants

A total of 94 refractory unilateral TLE patients (53 left and 41 right) were included in this study. A combination of EEG, MRI, PET, and neuropsychological testing was used to lateralize the side of seizure focus[25]. All patients met the following criteria: unilateral temporal lobe seizure onset through surface video/EEG recordings; normal MRI or MRI evidence of a temporal abnormality in the epileptogenic temporal lobe (mostly mesial temporal sclerosis (MTS), see Table 1); concordant PET finding of hypometabolism in the ictal temporal lobe. Patients were excluded from the study for any of the following: previous brain surgery; extratemporal or multifocal epilepsy; medical illness with central nervous system impact other than epilepsy; contraindications to MRI; drug abuse; psychiatric diagnosis other than an Axis-I Depressive Disorder; or hospitalization for any Axis I disorder listed in the Diagnostic and Statistical Manual of Mental Disorders, IV. Depressive Disorders were allowed given the high co-morbidity of depression and epilepsy [26].

**Table 1:** Demographic and clinical characteristics of the TLE patients and healthy controls.

**A.**								
	**Left TLE **			**Right TLE **			**Controls**	**p-value**
**N (females)**	53 (29)			41 (22)			50 (27)	*n*.*s*.^*¥*^
**Age—m (STD)**	40.5 (12.4)			40.5 (13.3)			39.4 (10.8)	*n*.*s*. ^*¥*^
**Age at TLE onset (years)**	21.7 (13.3)			22.3 (13.0)			-	*n*.*s*. ^*¥*^
**TLE duration (years)**	17.7 (14.2)			18.5 (14.1)			-	*n*.*s*. ^*¥*^
**Presence of structural abnormalities**	None (nTLE): 25			None (nTLE): 20			-	*n*.*s*. ^*¥*^
	mesial pathology (mTLE):			mesial pathology (mTLE):			-	*n*.*s*. ^*¥*^
	- MTS: 26			- MTS: 18				
	-mesial low grade tumor (e.g ganglioglioma, cavernoma): 2			-mesial low grade tumor (e.g DNET, cavernoma): 3				*n*.*s*. ^*¥*^
**Seizure Types**	CPS only: 19			CPS only: 14				*n*.*s*. ^*¥*^
	GTCS only: 1			GTCS only: 0				
	SPS only: 0			SPS only: 1				
	CPS/SPS: 5			CPS/SPS: 6				
	CPS/GTCS*:9			CPS/GTCS*:5				
	CPS/rare GTCS**:11			CPS/rare GTCS**:5				
	CPS with 2ndary GTCS: 9			CPS with 2ndary GTCS: 11				
**B.**								
	**L nTLE**	**L mTLE**	**p-val (mTLE vs nTLE)**^**£**^	**R nTLE**	**R mTLE**	**p-val (mTLE vs nTLE)**^**£**^	**Controls**	**p-val (R. vs L. TLE)**^*¥*^
**LM I**	28 (9)	23 (10)	*n*.*s*.	31 (11)	30 (10)	*n*.*s*.	40 (11)^a^	p = 0.002
**LM II**	18 (7)	13 (7)	p = 0.023	23 (7)	19 (8)	*n*.*s*.	34 (10)^a^	p = 0.001
**CVLT Tot**	49 (12)	44 (11)	*n*.*s*.	48 (12)	48 (12)	*n*.*s*.	52.7 (12)^b^	*n*.*s*.
**CVLT LDF**	11 (3)	8 (4)	p = 0.005	11 (3)	10 (4)	*n*.*s*.	12.3 (4)^b^	*n*.*s*.
**FM I**	35 (6)	35 (5)	*n*.*s*.	35 (5)	36 (4)	*n*.*s*.	37.5 (5)^a^	*n*.*s*.
**FM II**	36 (6)	35 (5)	*n*.*s*.	35 (5)	35 (5)	*n*.*s*.	37.5 (5)^a^	*n*.*s*.

Part A. describes demographic and clinical characteristics of our experimental groups. Part B. shows the neuropsychological scores (mean (standard-deviation)). n.s.: non-significant (p>0.05). Abbreviations: CPS: Complex partial seizures, GTCS: Generalized tonic-clonic seizure, MTS: Mesial temporal sclerosis, SPS: simple partial seizures; LM: Logical Memory, FM: Facial Memory; CVLT: California Verbal Learning Test, LDF: long delay free score; Tot: Total scores (Sum of the trials 1 to 5).

*: 10 or more GTCS in lifetime

**: less than 10 GTCS in lifetime

^a^: Normative results obtained on healthy age-matched participants (bin = [35–44] years) [27].

^b^: Normative results obtained on healthy age- and gender-matched participants (bin = [30–44] years) (manual of the CVLT II).

^¥^ 2-sample t tests between the right and left TLE groups (mTLE and nTLE combined).

^£^ post-hoc tests: two-sample t-tests between the nTLE and mTLE patients, within either right or left TLE patients.

Of note, TLE patients with presence of unilateral mesial pathology will be referred to as mesial TLE (mTLE) while TLE patients without visible structural brain abnormalities (revealed on the T1, based on the radiologist’s report) will be referred to as nTLE. Because of previous investigations suggesting that mTLE and nTLE constitute different pathologies [28], we analyzed their data separately.

Fifty healthy controls were recruited in order to match the patient participants in age, gender and handedness. All controls were free of psychiatric or neurological disorders based on health screening measures. This study was approved by the Institutional Review Board for Research with Human Subjects at Thomas Jefferson University. All participants have provided a written informed consent. Of note, all participants were fully capable to understand and comprehend the instructions. There was no indication of comprehension or other problems that precluded them to provide consent. However, if a participant was unable to provide a written informed consent a next of kin or legally authorized representative consented on behalf of the participant.

### MRI Data Acquisition

All participants underwent Magnetic Resonance Imaging on a 3-T X-series Philips Achieva clinical MRI scanner (Amsterdam, the Netherlands) using an 8-channel head coil. A total of 5 minutes of a resting-state condition was collected. Anatomical and functional acquisitions were similar for all participants. Regarding the resting-state condition, the participants were instructed to remain still, keep their eyes closed but not fall asleep throughout the scan. Single shot echoplanar gradient echo imaging sequence acquiring T2* signal was used with the following parameters: 120 volumes, 34 axial slices acquired parallel to the AC-PC line, TR = 2.5 s, TE = 35 ms, FOV = 256 mm, 128×128 data matrix isotropic voxels, flip angle = 90°. The in-plane resolution was 2*2mm^2^ and the slice thickness was 4mm. Prior to collection of the functional images, high resolution T1-weighted images were collected using an MPRage sequence (180 slices, 256×256 isotropic voxels; TR = 640ms, TE = 3.2ms, FOV = 256mm, flip angle = 8°, voxel size = 1x1x1mm). Each EPI imaging series started with three discarded scans to allow for T1 signal stabilization.

### Preprocessing analyses of the anatomical T1 sequence

In order to preprocess each individual T1 sequence, we used the VBM8 toolbox, available in SPM8 (http://www.fil.ion.ucl.ac.uk/spm/software/spm8). In detail, all T1-weighted images were preprocessed using the standard “estimate and write” routine: the images were spatially normalized to the same stereotaxic space (MNI space, Montreal Neurological Institute-152) using the fast Diffeomorphic Anatomical Registration Through Exponentiated Lie Algebra, (DARTEL) algorithm, segmented into GM, white matter and cerebrospinal fluid, and non-linearly modulated (correcting for local volume changes during the normalization), and, lastly, smoothed with an isotropic Gaussian kernel of 8 mm. A test of quality was performed to observe homogeneity and co-registration between the data. Seven subjects (4 left TLE, 3 right TLE) showing a T1 with an overall covariance below two standard deviations were excluded.

### Preprocessing analyses of the resting-state fMRI data

Resting-state fMRI data were preprocessed using SPM8. Slice timing correction was used to adjust for variable acquisition time over slices in a volume, with the middle slice used as reference. Next, a six-parameter variance cost function rigid body affine registration was used to realign all images within a session to the first volume. Motion regressors were computed and later used as regressors of no interest. To maximize mutual information, coregistration between functional scans and the MNI305 template was carried out using six iterations and resampled with a 7^th^-Degree B-Spline interpolation. Functional images were then normalized and warped into standard space (MNI305) to allow for signal averaging across subjects. We utilized the standard normalization method in SPM8. All normalized images were smoothed by convolution with a Gaussian kernel, with a full width at half maximum of 8mm in all directions. Sources of spurious variance were removed through linear regression: six parameters obtained by rigid body correction of head motion, the cerebro-spinal fluid and white matter signals. Finally, the data were temporally filtering in the band [0.008–0.1] Hz[29]. Eight TLE patients’ resting-state data (three left and five right) were excluded from further resting-state statistical analyses because of severe head motion (superior to 3 mm or degrees) or because the normalization step did not produce good results related to signal loss.

### Neuropsychological tests

To investigate verbal episodic memory (EM) functioning, we used scores from the Logical Memory (LM) subtests of the Wechsler Memory Scale III (WMS-III)[27], in addition to the total learning (TOT) and long delay free (LDF) recall scores from the California Verbal Learning Test (CVLT) II[30]. To investigate non-verbal EM outcomes, we used the scores of the Face Recognition Memory (FM) subtests from the WMS-III[27]. Standard administration instructions were utilized (e.g., the delayed recall condition was administered approximately 30 minutes after the immediate recall). These tests were administered as part of the neuropsychological test battery given to all patients undergoing presurgical evaluation at the Thomas Jefferson Epilepsy Center. The Logical Memory subtest is a verbal test that requires the examinee to freely recall a story after it is read aloud by the examiner. The Face Recognition Memory subtest is a visual, nonverbal memory test that requires a yes/no recognition response after two-second exposure to a series of 24 faces. Recognition testing includes presentation of 24 distractor faces, not previously presented. This task is free from any visuomotor (graphic) reconstruction requirements. For the FM test, two scores were used: the immediate (FM I) and delayed recall (FM II) scores; the same two comparable scores were generated for the LM test (LM I, LM II). These variables refer to the number of items remembered correctly.

Because six TLE patients did not complete neuropsychological testing at our center and one right TLE patient had invalid neuropsychological results (e.g. low confidence in the validity of the results), analyses involving these cognitive measures were conducted on 38 right and 52 left TLE patients.

### Group analyses

#### Gray matter analyses

First, a one-way ANOVA was computed on the post-processed T1 images to detect GM volume differences between our experimental groups (controls, left TLE, right TLE). Age and gender were added as covariates of non-interest. Then, as a sub-analysis, we recomputed the ANOVA to investigate the differences between the patients showing mesial pathology (mTLE, right and left TLE separately) versus a normal brain (nTLE, right and left TLE separately), relative to the controls. Left and right TLE patients were analyzed separately and were never combined. Of note, we did not directly compare right and left TLE in order to avoid bias or confound caused by, one, the intrinsic left-right asymmetries of brain, or, two, the differential impact right and left TLE are known to have on brain structure and function (see Discussion section for more detail). For these sub-analyses, only 25 controls were included in order to match the sample size of our patient groups. All the analyses included an absolute threshold masking of 0.1. Statistical whole-brain differences were reported with an initial statistical threshold of p<0.0001, uncorrected and a minimum cluster size of 30 contiguous voxels.

#### Resting-state FC analyses

Next, from the results obtained at the least stringent threshold (e.g. p<0.0001 uncorrected), we extracted the regions showing significant GM volume loss in the patients, relative to the controls (mTLE and nTLE, right and left TLE, considered separately). The resulting regions were used as regions of interest (ROIs) for the resting-state analyses. The goal was to be maximally sensitive to regions that might be part of an epileptic network, in investigating the FC between the regions showing significant GM volume differences versus controls.

In detail, an individual correlation map was produced by extracting the average BOLD time course from each ROI and then computing the correlation between that time course and the time course from all other brain voxels. Next, this map was submitted to a Fisher r-to-z transformation. For each region, two-sample t-tests were computed to identify differences in FC between the groups (e.g., controls vs. right mTLE, controls vs. right nTLE, controls vs. left mTLE). Statistical whole-brain differences were reported, with the height threshold fixed at p<0.0001 (uncorrected) and the spatial extent consistent with the expected number of voxels per cluster was utilized (k>10).

Of note, we purposely kept a consistent whole-brain height threshold for all our analyses (p<0.0001 uncorrected for both VBM and Resting-state analyses) but distinct spatial extent between the VBM and Resting-state analyses. The reason for this is that the resolution of VBM and resting-state data are quite different (voxel size = 1mm^3^ for VBM vs. 2 mm^3^ for Resting-state; an eight- fold difference). While previous studies (see review by [1]) have suggested that a stringent threshold (e.g. by applying a family-wise error correction) is highly recommended in VBM analyses, we compromised on the correction (not applying a whole brain correction but a stringent p: p<0.0001 instead of a typical p<0.001) in order to be sensitive to the resting-state data differences.

As a sub-analysis, we focused specifically on the FC solely between the regions with significant GM reduction (regions extracted from the initial ANOVA described below). In this case, two sample t-tests were computed between the patient and control groups (e.g., Left TLE versus Control; Right TLE versus Control; Left mTLE versus Left nTLE; Right mTLE versus Right nTLE). Significant differences were reported at p<0.05 corrected for the number of tests computed (based on the number of significant ROIs previously identified). Throughout the above analyses, right and left TLE and nTLE and mTLE were tested separately.

#### Analyses with the neuropsychological scores

Lastly, in order to measure the association between our functional and structural measures and the neuropsychological scores, we conducted linear regression analyses. Linear stepwise regression analyses were computed on the neuropsychological data (dependent variables tested separately: CVLT-TOT; CVLT-LDF; FM I; FM II; LM I; LM II) with the following as independent continuous variables (predictors): (1) the volume measure for GM ROIs found to be abnormal, (2) the pairwise FC (correlations) between GM ROIs found to be abnormal, (3) the interaction(s) between the effects involved in items (1) and (2), i.e., the abnormal GM volume and FC measures. The interaction(s) was inserted into the regression as a pre-multiplied product term (GM volume x FC measure). Each ROI was tested separately, and run separately within the right nTLE, right mTLE, left nTLE and left mTLE groups.

Lastly, for each significant result, we explored their relation with the age at TLE onset and disease duration.

The statistical analyses were run using SPSS (IBM Corp. Released 2011. IBM SPSS Statistics for Windows, Version 20.0. Armonk, NY: IBM Corp.).

## Results

### Behavioral data

The experimental groups did not differ in age nor gender (Table 1). Age at seizure onset or the rate of MTS was not significantly different between the patient groups. Regarding the neuropsychological data, the right and left TLE patients significantly differed for the LM test (LM I: p = 0.002; LM II: p = 0.001), with the left TLE showing lower scores. The patient groups did not differ on the other tests. When comparing their performance to normative values (provided in the manuals of the WMS-III[27] and the CVLT II for an age-matched healthy population, both right and left TLE patients showed reduced performance for the verbal episodic memory tests, but not for the non-verbal tests (FM I and II) (one sample t-tests against normative scores, p<0.05). When comparing mTLE versus nTLE patients, the CVLT II-LDF as well as the LM II scores were lower for the left mTLE patients (p = 0.023 and p = 0.005, respectively). There was no interaction between mesial pathology and pathology side for any of the neuropsychological tests. Also, there was no correlation between the age at TLE onset and any of the neuropsychological scores.

### VBM results

**Fig 1:**
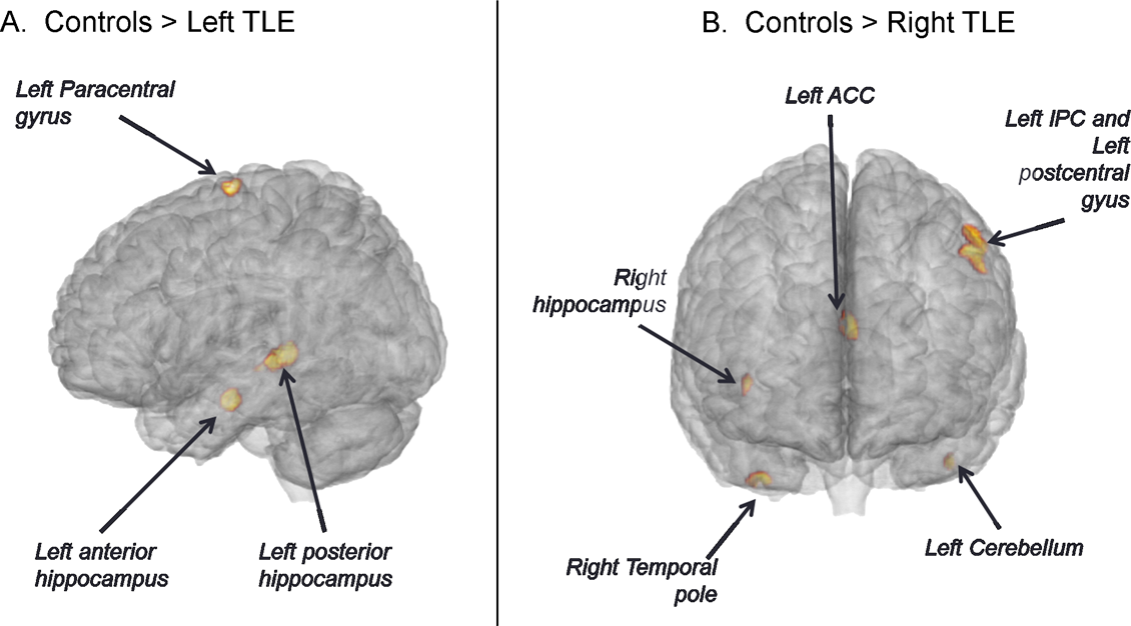
Regions with loss of gray matter in TLE patients. Left TLE in the left (panel A) and right TLE in the right (panel B).

Our analyses revealed several differences in regional GM volume between the three experimental groups (Table 2, S1 Dataset). Relative to healthy controls, the left TLE patients showed three regions with reduced GM volume located in the left paracentral lobule (PCL), and the anterior and posterior parts of the left hippocampus (Fig 1A). In contrast, they did not show any regions with an increase in GM volume, relative to the controls. When investigating the specific effects of MTS, we found that the left mTLE patients showed more extensive loss of GM, relative to the controls, than the left nTLE patients. The left mTLE patients had reduced GM in both anterior and posterior sections of the left hippocampus (Fig 1A). The left nTLE patients only showed reduced GM in a cluster located in the left cerebellum. No significant cluster was revealed in the ictal temporal lobe for the left nTLE patients.

**Table 2:** Voxel-Based Morphometric Results.

	Region	T	N voxels	x	y	z
**CTL—all LTLE**	L paracentral	5.36	112	-15	-12	75
	L Ant. Hippocampus	5.24	211	-32	-13	-20
	L PHG	4.83	526	-17	-36	-2
	L Post. Hippocampus	4.66		-18	-25	-6
	L Post. Hippocampus	4.33		-30	-31	-2
**All LTLE—CTL**	None					
**CTL—L mTLE**	L Ant. Hippocampus	6.46	432	-30	-15	-18
	L Post. Hippocampus	5.6	787	-18	-39	-2
	L Post. Hippocampus	5.06		-29	-31	-3
	L Post. Hippocampus	4.65		-18	-25	-6
**CTL—L nTLE**	L Cerebellum	4.68	204	-44	-64	-54
**CTL- all RTLE**	R Tp Pole	4.4	147	35	-4	-47
	L ACC	4.4	75	-2	38	25
	L Inf Parietal	4.4	94	-53	-27	43
	R Hippocampus	4.12	31	41	-22	-11
	L postcentral	4.07	44	-53	-16	52
	L cerebellum	4	33	-44	-66	-53
**All RTLE—CTL**	None					
**CTL—R mTLE**	R Hippocampus	6.18	1409	36	-12	-18
	R Tp Pole	5.06	482	24	-4	-48
	R Thalamus	4.35	361	11	-19	6
	R Postcentral	4.08	76	60	-3	24
**CTL—R nTLE**	L Inf Par	4.59	316	-53	-27	43
		4.41		-59	-21	34
	L Med Sup Frontal	4.57	341	-3	44	43
	ACC	4.43		0	35	30
	L postcentral	4.24	99	-47	-19	57
**L nTLE–L mTLE**	L Hippocampus	5.28	908	-30	-16	-17
	L Hippocampus	5.2		-26	-30	-3
**R nTLE–R mTLE**	R Hippocampus	6.12	1174	30	-13	-20
**LmTLE—LnTLE**	None					
**RmTLE—RnTLE**	None					

Group differences reported at p<0.0001 unc., k>50. Abbreviations: ACC: Anterior cingular cortex, Ant: anterior, Inf: Inferior, L: Left, Med: Medial, Mid: Middle, Par: Parietal, PHG: parahippocampal gyrus, Post: posterior, R: Right, SMG: supramarginal gyrus, Sup: superior, Tp: Temporal.

None of these structural abnormalities were correlated with either the age at TLE onset or the disease duration for the left mTLE group.

**Fig 2:**
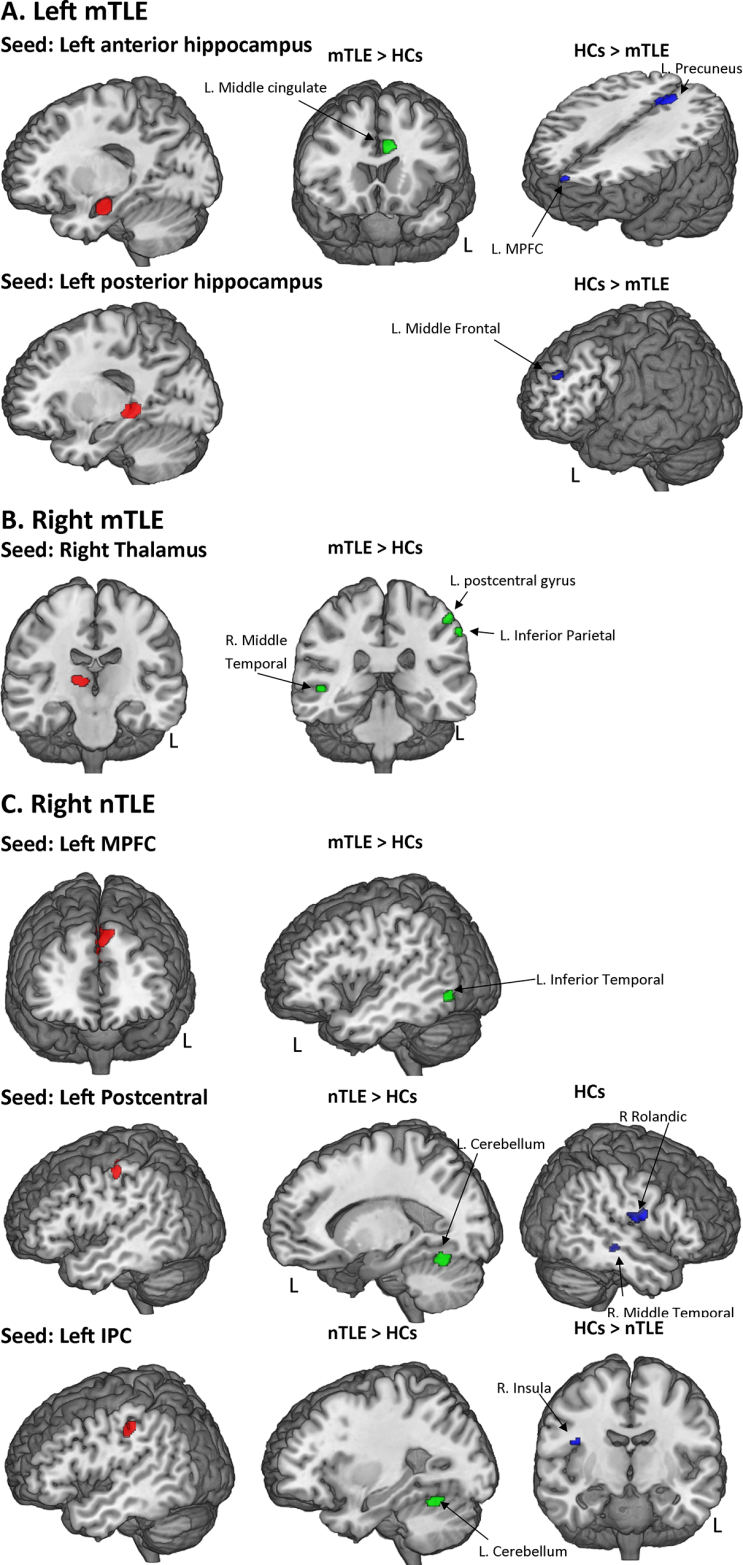
Regions with abnormal FC in TLE. Regions showing significant abnormal FC with each VBM-based ROI in the left mTLE (A), right mTLE (B) and right nTLE (C) patients, relative to the controls.

For the right TLE patients, we found reduced GM volume in six clusters located in the right hippocampus, the right temporal pole, the left anterior cingular cortex (ACC), the left inferior parietal cortex (IPC), the left postcentral gyrus and the left cerebellum relative to the controls (Fig 1B). When investigating the specific effects of MTS, we found that relative to controls the right mTLE patients showed more extensive loss of GM than the nTLE patients. The right mTLE patients had reduced GM in the right hippocampus but also in the right temporal pole, the right thalamus (Fig 2B, *left panel*) and the right postcentral gyrus. The right nTLE showed reduced GM in left-sided clusters in the IPC, postcentral gyrus, and medial superior frontal cortex (Fig 2C, *left panel*). While the age at TLE onset was not correlated with any of these clusters, structural abnormalities in the right hippocampus was negatively correlated with disease duration in the right mTLE group, indicating larger GM atrophy was associated with longer disease (r = -0.49, p = 0.025). Similarly, we found a negative correlation with disease duration and the degree of atrophy in the left postcentral cluster (r = -0.46, p = 0.039).

Overall, our analyses showed more GM volume loss in the ictal hemisphere, extending beyond the ictal temporal lobe, for all the patient groups except right nTLE. This extra-temporal GM loss appears more pronounced and regionally variable in the right TLE patients, as the loss for the left TLE patients loss is more strictly hippocampal and mesial. Also, our data demonstrate that mTLE patients have more GM volume loss than nTLE patients, especially in the ictal mesial temporal lobe. Finally, there was also an effect related to the side of pathology, suggesting a more pronounced reduction of GM volume in right compared to left TLE.

### Resting-state FC results

When seeding each ROI extracted from the VBM analysis, and interrogating the whole brain for significant correlations, we found the patients and controls displayed significant FC differences in a very limited set of regions (Fig 2, S1 Table). Note, because the left nTLE patients did not show regions with significant GM volume loss, relative to the controls, their data were not analyzed and are not reported in this resting-state section.

Regarding the left mTLE patients, the left anterior hippocampus (seed) showed reduced FC with both the left precuneus and left medial prefrontal cortex, relative to the controls. In contrast, it showed an increased FC with the left middle cingulate cortex, relative to the controls. Using the left posterior hippocampus as a seed only a small cluster in the left middle frontal cortex was evident with reduced FC, relative to the controls.

For the right mTLE group, the right thalamus was the only seed showing significant change in FC, with an increase, relative to the controls. This increased FC involved the left postcentral gyrus, the left inferior parietal cortex and the right middle temporal cortex.

For the right nTLE group, when seeding the left IPC or the left postcentral gyrus, the regions showing significant differences with controls were located in the right rolandic sulcus (more negative FC in the patients) and the left cerebellum (less negative in the patients). Lastly, the left medial prefrontal cortex was associated with less negative FC with a left inferior temporal cluster in the right nTLE patients, relative to the controls.

As a subanalysis, we explored the FC between the VBM-based ROIs, for each patient group separately (Table 3). We did not find any significant differences between the patient and the control groups, or significant correlation with either age at seizure onset or TLE duration.

**Table 3:** Functional connectivity between the regions showing reduced GM volume in the TLE patients.

**FC–Left mTLE**	**Patients**	**Controls**	**p-value**
**L Anterior Hippocampus—L Posterior Hippocampus**	0.33 (0.33)	0.33 (0.21)	*n*.*s*.
**FC–Right mTLE**	**Patients**	**Controls**	
**R Hippocampus–R Temporal Pole**	0.21 (0.28)	0.11 (0.24)	*n*.*s*.
**R Hippocampus–R Thalamus**	0.34 (0.28)	0.25 (0.22)	*n*.*s*.
**R Hippocampus—R Postcentral**	-0.08 (0.21)	-0.13 (0.21)	*n*.*s*.
**R Temporal Pole—R Thalamus**	0.07 (0.27)	0.02 (0.13)	*n*.*s*.
**R Temporal Pole—R Postcentral**	-0.09 (0.19)	-0.07 (0.18)	*n*.*s*.
**R Thalamus—R Postcentral**	-0.12 (0.17)	-0.21 (0.3)	*n*.*s*.
**FC–Right nTLE**	**Patients**	**Controls**	
**L IPC–L MPFC**	0.04 (0.17)	-0.04 (0.19)	*n*.*s*.
**L IPC–L Postcentral**	0.81 (0.29)	0.71 (0.39)	*n*.*s*.
**L MPFC–L Postcentral**	0.04 (0.15)	0.05 (0.28)	*n*.*s*.

FC (Z-values) correlation [mean (standard deviation)] between the regions showing reduced GM volume in the TLE patients compared to the controls. Abbreviation: ACC: anterior cingulate cortex; IPC: inferior parietal cortex.

Overall, our data indicated that while the abnormal GM regions of the patients showed some aberrant FC to regions elsewhere in the brain when examined individually, these regions as a set showed no abnormal inter-functional connectivity.

### Relation with neuropsychological scores

Using the GM volume and FC measures found to be abnormal relative to controls, we next sought to determine if these variables were reliable predictors of either verbal or non-verbal episodic memory performance. We utilized stepwise linear regressions to do so (Table 4).

**Table 4:** Results of the regression analyses predicting neurocognitive measures in the TLE patients.

**A. Left mTLE**			
**Verbal Measures:**	Adj R^2^	F(1,24)	P
**Model Effect for CVLT-LDF**	0.34	13.5	0.001
GM Volume in L. posterior Hippocampus		T = 3.7	0.001
**B. Right mTLE**			
**Non-Verbal Measures:**	Adj R^2^	F(2,17)	P
**Model Effect for FM I**	0.38	6.3	0.01
FC between R. Hippocampus and R. Thalamus		T = -2.4	0.027
FC between R. Temporal pole and R. Postc. G.		T = 2.2	0.047
**Model Effect for FM II**	Adj R^2^	F(1,17)	P
	0.2	5.3	0.035
FC between R. Temporal pole and R. Postc. G.		T = 2.3	0.035
**Verbal Measures:**			
**Model Effect for LM I**	Adj R^2^	F(1,18)	P
	0.2	5.6	0.03
GM Volume in R. Temporal pole		T = -2.4	0.03
**Model Effect for LM II**	Adj R^2^	F(1,18)	P
	0.17	4.8	0.042
GM Volume in R. Thalamus		T = 2.2	0.042
**Model Effect for CVLT TOT**	Adj R^2^	F(1,18)	P
	0.34	10.1	0.005*
GM Volume in R. Thalamus		T = 3.2	0.005
**Model Effect for CVLT LDF**	Adj R^2^	F(1,18)	P
	0.29	2.9	0.01
GM Volume in R. Thalamus		T = 2.9	0.01
**C. Right nTLE**			
**Non-Verbal Measures:**	Adj R^2^	F(1,14)	P
**Model Effect for FM I**	0.28	6.5	0.024
FC between L. IPC and L. MPFC		T = -2.2	0.047
**Verbal Measures**	Adj R^2^	F(1,15)	P
**Model Effect for LM II**	0.2	4.7	0.047
Interaction between GM Volume in L. Postc.G. and FC between L. Postc. G. and L. IPC		T = -2.2	0.047
**Model Effect for CVLT TOT**	Adj R^2^	F(1,15)	P
	0.25	6	0.028
Interaction between GM Volume in L. IPC and FC between L. Postc. G. and L. IPC		T = -2.5	0.028
**Model Effect for CVLT LDF**	Adj R^2^	F(1,15)	P
	0.24	5.5	0.036
Interaction between GM Volume in L. IPC and FC between L. Postc. G. and L. IPC		T = -2.3	0.036

Abbreviations: L = Left, R = Right, IPC = Inferior parietal Cortex, MPFC = Medial prefrontal cortex, Postc. G. = postcentral gyrus.

*significant at p<0.05, corrected for the number of tests done (n = 6).

**Fig 3:**
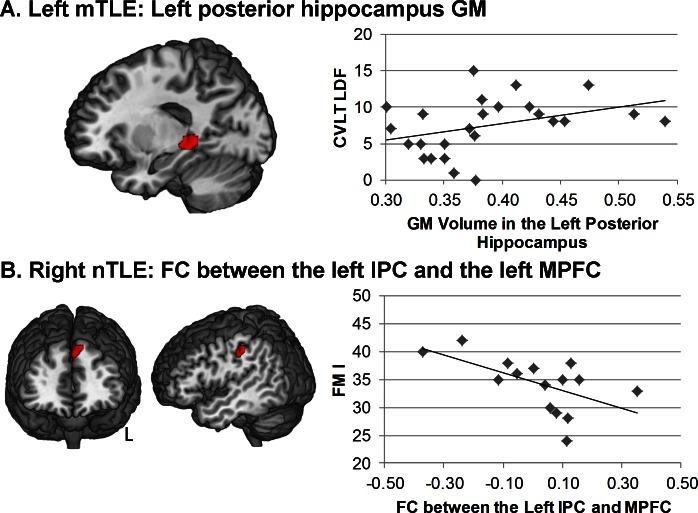
Relation between episodic memory scores and structural or functional measures in the TLE patients. A. Relation between the GM volume in the left posterior hippocampus and the CVLT-LDF score in the left mTLE. B. Relation between the FC between the left inferior parietal cluster and the left medial prefrontal cluster and the FM I Score in the right nTLE.

For left mTLE patients, the CVLT Tot measure was only predicted by the GM volume of the left posterior hippocampus (p = 0.001, adjusted R-square = 0.34) (Fig 3A). Non-verbal measures were not predicted by any of our GM or FC variables in the regression model involving left mTLE patients.

In contrast, for the right mTLE patients, FM I (full model, adjusted R-square = 0.38, p = 0.01) was best predicted by the FC measures between the following ROIs: right hippocampus and the right thalamus (p = 0.027) and the right temporal pole/right postcentral gyrus (p = 0.047). Similarly, FM II was best predicted by the FC measures involving the right temporal pole and the right postcentral gyrus ROIs (adjusted R-square = 0.2, p = 0.035). Lastly, for the right nTLE, FM I was best predicted by FC between the left inferior parietal cortex and the MPFC ROIs (adjusted R-square = 0.28, p = 0.024). The verbal measures LM I and LM II were only predicted by the GM volume of the right temporal pole ROI (p = 0.03) and right thalamus (p = 0.042), respectively. In the right mTLE, the CVLT TOT and CVLT LDF verbal memory measures were predicted by the GM volume of the right thalamus (effect p values of 0.04, 0.005 and 0.01, respectively).

For the right nTLE, FM I was best predicted by the FC measures involving the left IPC and the left MPFCC (adjusted R-square = 0.28, p = 0.024) (Fig 3B). In the right nTLE, both the CVLT TOT and CVLT LDF verbal memory measures were predicted by the interaction between the GM volume of the left IPC and the FC between the left post-central gyrus and left IPC (effect p values of .028 and .036 respectively). Also, the LM II was predicted by the interaction between the GM volume of the left postcentral gyrus and the FC between this ROI and the left IPC (effect p value of 0.047) (Fig 3B).

Thus, interaction effects between our structure (GM) and FC measures appeared only in models involving the right nTLE group. A consistent pattern in these interactions was the coupling of a single pair of left hemisphere nodes (left postcentral gyrus and left parietal cortex) with a posterior region of GM matter loss in the non-ictal, left hemisphere. Both forms of verbal memory recall (LM and CVLT LDF) were involved in these interactions. In each case, lower levels of memory recall were associated with higher levels of GM volume and increased FC in the relevant regions (noted above).

Overall, our results revealed few predictive indices for episodic memory in left mTLE, and in no case was a FC measure of predictive value. FC measures bore some predictive value for non-verbal memory, and GM measures some value for verbal memory measures, in the setting of right mTLE. A coupling of GM structure and FC was only evident in the setting of right nTLE. Here, a very limited but common set of non-ictal regions appeared predictive.

## Discussion

The present study identified whole-brain changes in GM volume associated with unilateral TLE, and examined how these changes related both to resting-state FC emerging from these abnormal regions and episodic memory performance. With our large sample of unilateral TLE, we also investigated the effect of the side of the pathology and the presence of MTS, throughout utilizing both verbal and non-verbal memory measures to keep track of material-specific effects.

Our VBM analysis revealed major GM changes in TLE compared to controls involving losses, mostly but not solely, in the ictal hemisphere, more prominent for the mTLE patients. Indeed, when directly comparing the mTLE to the nTLE, the major structural differences appeared in the ictal hippocampus with larger atrophy for the mTLE. These results are consistent with previous studies showing that nTLE patients show less, or even no structural abnormalities, relative to the mTLE patients[11]. McCormick et al. [21] also found that TLE was associated with GM volume loss primarily, though not exclusively, within the medial and lateral aspect of the epileptic temporal lobes. While the ictal mesial temporal lobe was the region showing the greatest reduction in GM, other extra-temporal regions were also significantly impacted such as the left frontal (paracentral) cortex and cerebellum, for left TLE, and the right thalamus, left cortical areas, as well as the cerebellum, for right TLE patients, relative to the controls. While the thalamus has been consistently described as atrophic in this population, and considered by some to be part of the TLE epileptogenic network[31], the role of the cerebellum remains unclear in epilepsy. There are some indications this cerebellar loss may be related to the iatrogenic effect of anti-convulsant medication[32]. It is also important to note that such cerebellar loss is concordant with previous VBM[10,12], single photon emission computed tomography (SPECT)[5], and FC studies in TLE describing abnormalities in the cerebellum [33,34].

Our data revealed major structural differences between left and right TLE, relative to the controls, with the former demonstrating more localized differences while the latter showing more widespread, bilateral changes. Such differences have been described by other studies, supporting the possibility that right and left TLE have a different impact on structural [35–37] organization across the hemispheres. It is important to note that in contrast to our results, there are studies [38] that have reported similar morphologic gray matter profiles for right and left TLE patients. Such studies, however, have involved Engel class 1 outcomes (seizure free patients) with multi-year follow-up. In pre-surgical samples, right and left TLE differences in gray matter are more commonly described. However, the extent of the laterality effect on the brain (e.g. localized versus widespread, relative to left versus right TLE) is not clear among all these studies, suggesting that our findings are specific to our sample rather than generalized. Certainly, more investigations need to be conducted to clarify in more detail GM integrity in the context of seizure onset laterality. Our results certainly suggest that right and left TLE bear distinct gray matter imprints, not just in terms of ictal region effects, but elsewhere in the brain, outside the temporal lobe, regardless of the presence of MTS. The reason for this remains unclear. One speculative possibility is that left versus right hemisphere TLE show differential spread patterns and, therefore, potentially different levels of gray matter burden. However, a recent study and review of spread pattern studies in TLE provided no indication of such, though they did not specifically investigate spread differences as a function of left versus right hemisphere seizure generators [39]. Alternatively, hemispheric language dominance may play a role, possibly providing an example of how functionality can influence and alter structural resiliency or neural reserve[14,16].

Importantly, we tested the hypothesis that the abnormal GM regions noted above would be associated with abnormal FC, potentially pointing to the presence of an existing or emerging epileptic network entrained and generated by seizure spread or epileptogenesis [13,40]. In contrast, we found that compared to controls these structurally abnormal regions did not display abnormal inter-connections (i.e., FC) amongst themselves (i.e., their FC connections were similar to matched controls at the time we scanned them, see Table 3). This does not imply that these areas bore no FC, only that such connectivity was not abnormal compared to our normative reference group. This also does not preclude these regions from generating functional connections, perhaps quite different from normal controls, under other conditions. While our finding does seem to argue against the notion that structurally abnormal regions tend to form epileptiform networks, other factors need to be considered. It is also possible, for instance, that resting-state FC measures are insensitive to seizure network effects due to differences in the time scale of fast, hypersynchronous seizure activity in cells, and the slower, longer time course of BOLD/neuronal coupling [40,41]. It is also important to keep in mind that we did not test for indirect (e.g., polysnaptic) connections between the atrophic regions, which could explain the lack of evidence for functional communication between gray matter areas that seem to share a structural abnormality (i.e., gray matter atrophy from seizures). Another alternative possibility is that these GM regions are part of an ictal epileptic network, but such network formation was not evident in the inter-ictal state during which our resting-state scans were collected. Such an interpretation is consistent with recent SPECT studies that have shown differences in epileptic network formation between ictal and interictal states in TLE[4,5]. For instance, Sequeira et al. [4] found the pattern of connectivity differs between the two states, with decreased correlations between the epileptogenic temporal lobe and remaining cortex during the interictal state, but emergent positive cross-correlations in temporal-limbic structures during the ictal phase of seizures. In fact, these authors proposed that there is a relative disconnection between the epileptogenic temporal lobe and the rest of the brain during inter-ictal periods.

In comparison to other studies examining links between gray matter structure and resting state FC, our results reveal that in focal TLE seizure spread may affect multiple FC networks rather than just one. In this regard, we should note that in both our structural and FC data, for the right mTLE patients, the thalamus emerged as a common area, raising the possibility that damage to this structure, a structure known to play a role in seizure generalization, may play a role in perturbing resting FC networks. For instance, as we noted previously, McCormick et al. [21] solely addressed the association between focal structural damage in the epileptic hippocampus specific patterns of DMN FC alterations. Our focus was broader, allowing for any extra-temporal GM abnormality to emerge. While some of the regions we found to have GM loss in our TLE patients were part of the DMN, the majority were not, and in fact, were anti-correlated to this network [42,43]. As others have noted, the structural substrates for observed functional connectivity differences are complex, and the latter cannot be assumed to reflect the former [44]. Voets and colleagues [45], for instance, found reduced functional integration of the hippocampus was associated with variability in gray matter structure, whereas functional connectivity of nearby regions such as the parahippocampal gyrus, frontal, or temporal cortices appeared instead to be associated with white matter structure abnormalities. These authors discussed methodological confounds (e.g., gray/white matter blurring, gray/white matter measure co-linearity) behind such variability in structure/function relationships. Importantly, their data and ours make clear that function-structure relationships in TLE are highly sensitive to the analysis strategy and statistical method used. Further investigations will certainly need to be conducted to explore in more detail GM and FC associations in TLE, determining if a more generalizable pattern is present and verifying the direction of any effects (e.g., does gray matter atrophy increase or decrease functional connectivity).

Given that our VBM-based ROIs were chosen on the basis of volume loss most likely inflicted during the ictal rather than interictal state, our failure to find compelling evidence of inter-ictal functional connectivity suggests that ictal and inter-ictal functional connectivity patterns may be quite different, or that the abnormal regions we examined are actually part of distinct epileptic networks. However, one must consider that our choice of VBM-based ROIs may have been biased, for instance, by our choice of smoothing kernel or whole-brain threshold. Another approach would have been to base our FC analyses on regions defined by an anatomical atlas. Since such an approach has been extensively used in other resting-state papers [15,46,47], we chose to use a different method to investigate the link between structural and functional abnormalities in TLE patients. Overall, multi-modal investigations, sensitive to different network states, time scales and spatial resolutions, working to combine and integrate both ictal and inter-ictal data, will be needed to fully confirm and characterize epilepsy-related connectivity patterns.

In our FC analyses, we also utilized our VBM-based ROIs as single seeds and examined connectivity to the whole brain. In the case of left mTLE, as might be expected, the aberrant networks compared to controls did, by and large, emerge from the ictal region (left anterior hippocampus). Our data also make clear that the extra-temporal regions involved, i.e., potentially recruited into this network, were mostly ipsilateral in nature involving the left precuneus and medial prefrontal cortex. These regions are part of the well-known DMN, and, in fact, are defined as the key DMN hubs[48,49]. Our lab and others have described the DMN as abnormal in TLE patients, especially for left TLE patients [15,21,50,51]. Therefore, our results are consistent with previous literature, further confirming that the DMN is particularly affected by left TLE seizures. However, it is important to highlight another potential implication of reduced precuneal FC in our left TLE group. That is, it may reflect a more general process of seizure spread and its associated pathology impacting high traffic areas of the brain, hubs, before burdening other less trafficked brain nodes.

In contrast, for right TLE, the connectivity differences compared to controls involved ROIs such as the ipsilateral thalamus, temporal cortex, the medial frontal and the left parietal cortices, demonstrating numerous connections involving contralateral sensory-motor regions. As noted, the thalamus was among the regions associated with both structural and functional abnormalities, confirming its key role in both the structural and functional network effects of even focal TLE. For this group, our finding of abnormal FC in sensory-motor regions adds to the likelihood that our data have revealed the multiple independent networks revealed by seizures. While unexpected, a previous study has indicated that sensory and perceptual networks are impaired in TLE[52]. For both our left and right TLE groups it is important to note that the instances of reduced connectivity involve mostly connections ipsilateral to the seizure focus. In the cases of increased FC, however, our groups differed, with the left group showing mostly ipsilateral abnormalities, and the right group showed mostly contralateral examples of abnormally increased FC. Why the FC effects of right TLE would be more prone to a contralateral impact is unclear. Similarly, it is not clear why we did not reveal FC abnormalities emerging from the right epileptic hippocampus. This might have been caused by our stringent height whole-brain threshold.

As was the case for understanding the greater extra-temporal GM volume loss in our mTLE patients a more extensive pattern of seizure spread may be at work, or the impact of the most crucial functional difference between the hemispheres (language dominance) may be altering the resiliency or durability of node temporal synchrony during the resting state.

Regarding the relation between our structural and functional measures and episodic memory scores, we found that the left TLE patients produced fewer reliable effects, in fact, only a single significant effect, material specific in nature, involving reduced verbal memory recall and decreased GM volume in the left posterior hippocampus. This finding is not new to the literature [21,24,53] and, more notably, is consistent with literature showing that different regions of the mesial temporal lobe bear distinct relationships with the component processes involved in episodic memory (posterior/lateral areas mediating pattern generalizing item-context representations support familiarity/recognition memory, and the anterior hippocampus mediating pattern specific item memory involved in free recall)[38,53–55].

Our findings for right mTLE show that GM volume loss in right-sided structures is associated with reduction in verbal memory problems, both at the point of acquisition and recall. Interestingly, in right mTLE, FC only showed associations with non-verbal memory. The reasons for these distinct patterns for our FC and structural measures, are unclear. In contrast, right non-lesional TLE was most commonly associated with weaker verbal memory, both acquisition and retention, but only when there was a coupling (i.e., interaction) of our structural (GM) and functional (FC) measures involving the non-ictal hemisphere. Said differently and more specifically, increased GM in certain non-ictal extra-temporal areas (postcentral or inferior parietal), appeared to be predictive of reduced verbal memory but only in the setting of increased FC involving these regions. This suggests that in occult, non-lesional pathology both structural and functional alterations may be necessary to produce cognitive deficits.

As to why the structure/function coupling we observed in right nTLE consistently produced connections involving the postcentral gyrus and inferior parietal cortex remains unclear. These associations, while unexpected, have been described in the literature, with a previous study indicating that sensory and perceptual networks are, indeed, impaired in TLE [52], as part of its extra-temporal impact [40]. With regard to right nTLE, it is again important to note both that the instances of increased functional connectivity were contralateral to the seizure focus, associated with increased GM matter, and predicted reduced episodic memory. Accordingly, this extra-temporal coupling of structure and function appears maladaptive, and limited to non-lesional (and probable non-dominant hemisphere) TLE. In contrast, by our data, lesional TLE does not produce these more widespread maladaptive effects.

It is important to note that our neuropsychological analyses were undertaken only to clarify the potential cognitive and behavioral implications of a very select set of regions, chosen solely on the basis of their demonstrating abnormal structure/function relationships. Thus, our neuropsychological data neither re-test nor contradict previous work showing relationships between memory and regional FC when considered in the absence of concurrent structural (i.e., GM) abnormality. In other words, the neurocognitive relationships we observed do not preclude the emergence of more standard memory/GM or memory/FC results, if they are undertaken through standard whole brain analyses without the use of pre-selected regions.

Lastly, it should be noted that our patients may have been misclassified between mTLE and nTLE, and therefore, biasing the results. While the classification of the mTLE patients was based on multiple anatomical sequences and careful review by an Epilepsy Surgery Committee including radiologists, neurologists, neurosurgeons, and neuropsychologists, all specialized in epilepsy, misclassifications remain possible.

To our knowledge, this study is among the first lines of evidence to suggest that episodic memory capacity in left and right TLE are associated with distinct structural and functional measures. We acknowledge, however, that our findings in support of this interactive explanation of our cognitive data are limited, and, in fact, only appeared for non-lesional right TLE cases. Indeed, the fact that seeding both the hippocampus and other perturbed GM regions did not produce networks relevant to episodic memory (or cognitive functions), makes the point that neither seizure-generated networks, nor the location of seizure-related GM abnormalities, need make sense in either adaptive or cognitive terms. Accordingly, while we had suspected episodic memory deficits might be influenced by both structural and functional abnormalities, our data suggest they show some independence in terms of a causal chain. Lastly, as a caveat, with respect to neurocognitive performance, our FC analyses were not exhaustive. For instance, as we did not specifically test the connectivity status of a known episodic memory network (i.e, the default mode network, like [21]), nor other brain networks that could influence episodic memory performance (e.g., executive function), nor did we utilize all possible indices of network integrity in our interrogation of abnormal GM connectivity. Ultimately, the exact mechanism by which structural and functional connectivity abnormalities might combine to mediate memory and other cognitive deficits in TLE remains unclear and in need of further study.

## Conclusion

This study investigated abnormalities in refractory TLE using a multi-modal approach combining GM, FC and neuropsychological measures. Our data revealed distinct patterns of structural abnormality in TLE patients, which varied as a function of the side of pathology and the presence of MTS. Importantly, we did not find that the GM abnormalities were functionally or abnormally connected during the resting state. This result is consistent with the possibility that the atrophic regions we tested do not form a functional epileptic network. Alternatively, one must consider that our results simply reflect the weak sensitivity of functional connectivity in terms of detecting epileptic networks during the inter-ictal state. We provide evidence of the role that atrophy in the ictal posterior hippocampus plays in episodic verbal memory in the setting of left mTLE. We also show that in the setting of right hemisphere lesional TLE, FC and structural measures abnormalities, ipsilateral to the seizure focus, produce distinct material-specific effects on memory. Lastly, we provide data suggesting that in the setting of occult, non-lesional right TLE pathology, a coupling of structural and functional abnormalities in extra-temporal/non-ictal regions is necessary to produce reductions in episodic memory recall. The latter, in particular, demonstrates the complex structure/function interactions at work when trying to understand cognition in TLE, suggesting that subtle network effects can emerge bearing specific relationships to hemisphere and the type of pathology.

## Supporting Information

S1 Table Regions showing significant differences in FC between controls and left mTLE (A), right mTLE (B), or right nTLE (C) when seeding the ROIs extracted from the VBM analysis (T>3.9).
(DOC)

S1 Dataset 2 nifti files, including the whole-brain comparison between each patient group and the controls for the VBM analysis (Controls—Right TLE and Controls–Left TLE). The clusters in each file are described in Table 2 and illustrated on Fig 1.
(ZIP)
