# Supplementary material for: Gray Matter Abnormalities in Temporal Lobe Epilepsy: Relationships with Resting-State Functional Connectivity and Episodic Memory Performance
Source: PLoS One. 2016 May 12;11(5):e0154660. doi: 10.1371/journal.pone.0154660 (PMC4865085; doi:10.1371/journal.pone.0154660)
Supplement: S1 Table — Regions showing significant differences in FC between controls and left mTLE (A), right mTLE (B), or right nTLE (C) when seeding the ROIs extracted from the VBM analysis (T>3.9). (DOC) [file pone.0154660.s001.doc]

**Supporting Information**

**Gray matter abnormalities in temporal lobe epilepsy: Relationships with resting-state functional connectivity and episodic memory performance**

Gaelle E. Doucet, XiaoSong He, Christopher Skidmore, Ashwini Sharan, Michael Sperling, Joseph Tracy

**S1 Table:** Regions showing significant differences in FC between controls and left mTLE (A), right mTLE (B), or right nTLE (C) when seeding the ROIs extracted from the VBM analysis (T>3.9).

| **A. Left mTLE** | **L Anterior Hippocampus** | **L posterior Hippocampus** |
| --- | --- | --- |
| **Controls - Left mTLE** | L Precuneus  (-4,-52,44; T=5.1; k=196) | L Mid. Frontal (-30,46,32; T=4.6; k=17) |
|  | L MPFC  (-8,68,14; T=4.6; k=16) |  |
| **Left mTLE - Controls** | L Mid. Cingulate  (-10,14,28; T=5.4; k=66) | *None* |

| **B. Right mTLE** | **R Tp Pole** | **R. Thalamus** | **R. Hippocampus** | **L Postcentral gyrus** |
| --- | --- | --- | --- | --- |
| **Controls - Right mTLE** | *None* | *None* | *None* | *None* |
| **Right mTLE - CTL** | *None* | L Inf. Parietal (-58,-36,42; T=5.6; k=50) | *None* | *None* |
|  |  | R Mid. Temporal  (46,-32,-2; T=4.7; k=17) |  |  |
|  |  | L postcentral (-50,-30,52; T=4.7; k=23) |  |  |

| **C. Right nTLE** | **L. Medial prefrontal cortex** | **L. Inferior Parietal Cortex** | **L Postcentral gyrus** |
| --- | --- | --- | --- |
| **Controls - Right nTLE** | *None* | R Insula (38,-8,20; T=5.5; k=15) | R Rolandic (42,-8,20; T=6.1; k=116) |
|  |  |  | R Mid. Temporal (52,-28,-8; T=4.8; k=28) |
| **Right nTLE - CTL** | L Inf. temporal (-54,-64,-9) | L cerebellum (-28,-58,-20; T=4.9; k=42) | L cerebellum (-28,-56,-18; T=5; k=54) |

Abbreviations: L: Left, R: Right, MPFC: Medial Prefrontal Cortex, mid: middle, inf: inferior.
